# The Influence of Fly Ash Carbonation on the Protective Properties of Concrete Cover Towards Reinforcement

**DOI:** 10.3390/ma18102181

**Published:** 2025-05-09

**Authors:** Beata Jaworska, Dominika Stańczak, Rafał Kobyłka, Tomasz Gołofit, Duo Zhang, Justyna Kuziak

**Affiliations:** 1 Faculty of Civil Engineering, Warsaw University of Technology, Armii Ludowej 16, 00-637 Warsaw, Poland; dominika.stanczak@pw.edu.pl (D.S.); justyna.kuziak@pw.edu.pl (J.K.); 2 Institute of Agrophysics, Polish Academy of Sciences, Doświadczalna 4, 20-290 Lublin, Poland; r.kobylka@ipan.lublin.pl; 3 Faculty of Chemistry, Warsaw University of Technology, Noakowskiego 3, 00-664 Warsaw, Poland; tomasz.golofit@pw.edu.pl; 4 School of Water Resources and Hydropower Engineering, Wuhan University, 299 Bayi Road, Wuhan 430072, China; duo.zhang@whu.edu.cn

**Keywords:** CO_2_ sequestration, calcareous fly ash, combustion byproduct, carbonation, chloride ions

## Abstract

To address the challenge of reducing carbon dioxide emissions, this study focuses on carbon dioxide sequestration in calcareous fly ash and its use in mortar and concrete specimens, including reinforced structures. Calcareous fly ash was used in this study because it contains more reactive Ca phases, enabling efficient CO_2_ capture and long-term storage through mineral carbonation. The research examines the influence of incorporating carbonated fly ash on the protective properties of the concrete cover for steel reinforcement, along with an analysis of the mechanical behavior of the specimens, resistance to weathering carbonation, and the modeling of the service life of reinforced concrete structures. The results indicate that the compressive strength of concrete specimens decreases with the addition of carbonated ash, though by no more than 9% after 90 days. The carbonation rate of concrete increases with the addition of ash; however, a roughly 4% lower rate was observed for carbonated ash compared to non-carbonated ash. No significant impact of ash carbonation on chloride diffusion or the corrosion process of reinforcement in mortars was detected. As a result, the estimated service life of concrete containing both ash and carbonated ash is over 20 times longer than that of the reference concrete.

## 1. Introduction

The swift advancement of worldwide industries has resulted in the widespread usage of fossil fuels, contributing to a significant increase in atmospheric CO_2_ concentrations. The CO_2_ emissions of Poland reached 303.5 mln tons in 2020, where cement production accounts for 6.3% and energy industry for 45.8% of it [1]. Such large CO_2_ emissions affect global warming and may cause irreversible changes on the Earth. In order to prevent those changes, a strategy of capturing, utilizing, and storing carbon dioxide (CCUS) has been introduced [2,3]. There are three main methods for storing CO_2_, which encompass ocean storage, geological storage, and mineral storage, with mineral storage being the most commonly used in the building industry. Mineral carbonation is a widely recognized method for storing carbon, first suggested by Seifritz in 1990. It involves the chemical process of mixing materials rich in metal oxides like CaO and MgO with CO_2_ to produce carbonates that are not soluble in water [4]. There are two primary pathways during mineral carbonation: the dry and the wet process of carbonation [5]. Mineral carbonation can make use of various raw materials, such as calcium-, magnesium-, and iron-rich silicates as well as mining waste, steel by-products, ash from combustion, and recycled concrete aggregates (RCA) [5,6], and its CO_2_ sequestration potential capacity was estimated at 100–250 × 1012 tons [6]. The effectiveness of CO_2_ capture is significantly influenced by the quantity of CaO and MgO present in the materials.

While carbonation curing has been proposed as a way of curing cement-based materials with CO_2_, its implementation in reinforced concrete faces challenges due to lowered alkalinity. When the concrete pH drops below 11.8, a passive layer does not form on the reinforcing steel, leading to steel corrosion [7,8]. This highlights the critical relationship between carbonation, concrete pH, and the onset of corrosion in reinforced concrete structures.

Incorporating supplementary cementitious materials (SCMs) is proven to be an effective approach for developing low-carbon cement. Research has indicated that the utilization of SCMs as a partial substitute for Portland cement (PC) can result in a decrease in CO_2_ emissions by 6.0% [9]. As a typical SCM, coal fly ash has the potential of sequestering CO_2_ through mineral carbonation, thus leading to additional emission reduction by utilizing CO_2_ in concrete [10,11]. Particularly, class C fly ash contains a high amount of CaO, which makes it an attractive material for CO_2_ sequestration in the form of stable CaCO_3_. Calcareous fly ash (CFA), due to its high calcium content in the form of free lime (CaO) and calcium silicates, is more reactive with CO_2_ than siliceous fly ash, making it an ideal candidate for CO_2_ sequestration through mineral carbonation. This higher reactivity facilitates the formation of stable CaCO_3_, which locks away CO_2_ in a solid, stable form, thereby contributing to long-term carbon storage. In contrast, siliceous fly ash, which contains primarily silica-based minerals (SiO_2_), shows lower reactivity and is therefore less effective for CO_2_ sequestration [12].

Studies have demonstrated that fly ash has a CO_2_ storage capacity of up to 7.28 g CO_2_/100 g [13]. Siriruang et al. investigated the use of fly ash as a solid sorbent for CO_2_ capture and then as an additive in cement mixtures after the CO_2_ capture process [14]. There are no significant differences in the compressive strength in specimens using fly ash before and after CO_2_ capture. When using 20 wt.% of fly ash after 91 days of curing, specimens containing carbonated ash were characterized with 4% lower compressive strength compared to specimens with non-carbonated ash. The fly ash, after CO_2_ capture, as the mineral additive can slightly decrease the carbonation resistance in mortar specimens. Compared to cement-only mortar, the carbonation depth of all mortar specimens using 30 wt.% fly ash replacements was almost 100% deeper than that of cement-only mortar. The results also show that mortar specimens using fly ash after CO_2_ capture have 9% deeper carbonation depth than those using fresh fly ash in all batches of fly ashes. Research conducted by López-Zaldívar et al. [15], Andrew et al. [16], and Pei et al. [17] also demonstrated that incorporating carbonated fly ash can enhance the strength of specific types of cement mortars. Another investigation that demonstrates the practicality of utilizing fly ash as a cement additive is the study conducted by Ebrahimi et al. [18]. In this study, carbonated fly ash (CCFA) was employed to create cement mixtures, where Portland cement (PC) was substituted with CCFA at levels of 10% and 30% (*w*/*w*), using a water-to-cementitious material ratio of 0.4. The carbonated fly ash exhibited a maximum carbon sequestration capacity of 3.8 g CO_2_/kg of adsorbent with a carbonation efficiency of 16.9%. Although the compressive strength of the samples declined as the CCFA content increased, the reduction was minimal at the 10% replacement level, indicating that the performance of the cement/CCFA mixture was comparable to that of pure cement.

However, the carbonation of fly ash affects the carbonation behavior of concrete, influencing the durability of reinforced concrete. The deterioration of rebars within reinforced concrete has been identified as a significant issue concerning the preservation of the structural integrity of buildings and other structures. The pH level of concrete, which typically ranges from 12 to 13.5, offers a chemical shield for the rebars by enabling the passivation of steel. Nevertheless, over time, substantial corrosion issues can arise in reinforced concrete structures. The primary factors that contribute to the onset of corrosion in reinforcing steel include the penetration of chloride ions and carbon dioxide onto the steel surface. Chloride ions can cause the breakdown of the passive film, leading to localized corrosion. In contrast, carbon dioxide interacts with the hydrated cement matrix, resulting in a reduction of pH, which subsequently causes a loss of steel passivity and triggers the onset of corrosion. In an earlier study [19], it was noted that the diffusivity of chlorides in submerged concrete was decreased to 50% of its original value when 50% fly ash was incorporated. As a result, the corrosion process caused by chlorides was significantly postponed. Similarly, incorporating 30% fly ash as a partial replacement for cement resulted in a marked extension of the induction period and a tenfold reduction in the corrosion rate [20]. In the context of carbonation, the inclusion of fly ash appears to have an opposing influence. It is well established that carbon dioxide interacts with Ca(OH)_2_ resulting in the formation of CaCO_3_. The calcium carbonate is insoluble and precipitates, effectively obstructing the pores in the concrete and slowing down the advancement of the carbonation front [21]. When part of the Portland cement is replaced with fly ash, the amount of calcium silicates is reduced, leading to less formation of Ca(OH)_2_ and, consequently, a decrease in the pH level of the concrete. Additionally, some of the Ca(OH)_2_ is consumed during pozzolanic reactions. With a lower concentration of Ca(OH)_2_ and as a result of this decreased pH, the movement of the carbonation front is quicker. Once the carbonation front reaches the reinforcing bars, the resulting neutralization causes the depassivation of the steel and triggers the corrosion process. Studies have confirmed that fly ash accelerates CO_2_-induced corrosion [20,22], underlying the need to balance the benefits of CO2 sequestration with the potential risk of corrosion in fly ash-containing concrete.

All of the aforementioned studies focus on CO_2_ sequestration in siliceous fly ashes and, separately, the carbonation of reinforced concrete samples containing fly ash.

A unique contribution of this study is its utilization of the carbonated calcareous fly ash (CFA) in concrete and reinforced mortar. Substituting 10 wt.% CFA for cement was selected for the preliminary tests, balancing the enhancement of CO_2_ uptake with the preservation of mechanical performance. This replacement level is consistent with common practices in concrete production, where it has been shown to offer an optimal combination of strength, durability, and carbonation resistance without significant degradation of structural properties [18]. To understand FA’s carbonation behavior, thermogravimetric analysis, X-ray powder diffraction, and scanning electron microscopy were conducted, while an electrochemical analysis was carried out on the reinforced specimens to assess the impact on reinforcement corrosion. Moreover, the chloride diffusion and weathering carbonation behavior of cement composites incorporating carbonated FA were investigated. The results were used to inform the service life prediction of the reinforced concrete and to determine the durability of reinforced concrete structures at risk of chloride ingress.

## 2. Materials and Methods

### 2.1. Mixture Design and Specimen Preparation

The calcareous fly ash (PGE Capital Group, Bełchatów Power Plant, Bełchatów, Poland) and Portland cement CEM I 42.5R (Górażdże, Poland), with their chemical composition shown in Table 1, were used in this study. The specific surface area, measured with the Horiba LA-300 laser particle size analyzer (HORIBA Scientific, Kyoto, Japan), is 15,063 cm^2^/cm^3^ for calcareous fly ash (P) and 20,039 cm^2^/cm^3^ for cement. Fly ash was characterized by density equal to 2.3 g/cm^3^ measured according to PN-EN 1097-7 [23] and by fineness equal to 51.2% measured in accordance with PN-EN 451-2 [24]. As seen in Table 1, the predominant oxides found within the sample of calcareous fly ash before (P) and after (PK) CO_2_ sequestration included SiO_2_, Al_2_O_3_, and CaO.

**Figure 1:**
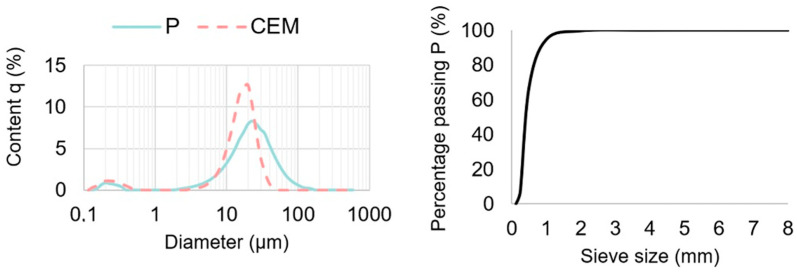
Grain size distribution curves for calcareous fly ash (P) and cement (CEM) and Vistula sand sieve analysis.

Table 2 shows the mix designed of mortars and concrete specimens used in this study. The grains size distribution analysis of fly ash and cement was made using Horiba L300 laser analyzer and is shown in Figure 1. For each material, three samples were tested. Calcareous fly ash (P) consisted of grains with diameters exceeding 100 µm, in contrast to cement (CEM), which was characterized mostly by grains no larger than 52 µm diameter. Both tested materials contained particles smaller than 4 µm and similar content of particles smaller than 45 µm (calcareous fly ash—89% and cement—99%). Average diameter size of calcareous fly ash particles was about 21 μm, only 2% smaller compared to cement particles. Gravel aggregate with a maximum size of 16 mm was used as coarse aggregate, and Vistula sand (Figure 1) was employed as fine aggregate in concrete specimens. Mortar specimens were prepared using normal sand according to PN-EN 196-1 [25] with quartz content above 98%. For all compositions, the water/cement ratio (w/c = 0.5) was assumed as a constant value. In order to attain good workability of concrete, a superplasticizer based on stabilized polycarboxylates was used. Despite using a constant w/c in all the concrete batches, the concrete mixtures were characterized with similar concrete slump, which was 220 mm for reference concrete (CR), 200 mm for concrete with the non-carbonated fly ash (CP), and 225 mm for concrete with carbonated fly ash (CPK). The slump test was performed after the addition of the superplasticizer. Concrete specimens were cast into 100 mm and 150 mm cubes. The former was used to test compressive strength and the rate of weathering carbonation, while the latter for measuring the diffusion of chloride ions. Mortars were also prepared in two types of molds, beams with the dimensions of 40 mm × 40 mm × 160 mm for the strength test, rate of weathering carbonation, pH determination, and cylinders with a diameter of 50 mm and a height of 150 mm, with steel reinforcement for the electrochemical test (electrochemical impedance spectroscopy (EIS) and measurements of anodic polarization curves).

### 2.2. CO_2_ Sequestration Process

The aim of the study was to analyze calcareous fly ash exposed to high-pressure carbonation for approximately 24 h and assess the feasibility of using carbonated fly ash as a replacement for cement in concrete production without significantly impacting the performance of the concrete. Slightly moistened calcareous fly ash was subjected to carbonation in a closed reactor, to which pure carbon dioxide was delivered under a pressure of 5 bar for 24 h. The carbonation conditions were optimized for laboratory testing to maximize CO_2_ sequestration while maintaining a balance between practical feasibility and process efficiency. Immediately after the CO_2_ sequestration process, samples of mortars and concretes were prepared, in which 10 wt.% carbonated calcareous fly ash was used as a substitute for cement.

### 2.3. CO_2_ Uptake Measurement

Fly ash has the ability to capture CO_2_ through two distinct processes: adsorption on the surface of a solid sorbent and by a carbonation reaction. In terms of adsorption, the capture of CO_2_ can take place through the physical adsorption process, involving the interaction between CO_2_ and the solid sorbent’s surface. Hence, the high surface area of a solid sorbent facilitates the presence of sites for CO_2_ adsorption [26,27]. Nevertheless, this process can be undone at temperatures exceeding 120 °C due to the solid sorbent’s ability to release the CO_2_ back into the atmosphere upon activation through heating [28]. In terms of the carbonation reaction, the presence of a sufficient amount of free CaO in the fly ash is crucial for this reaction. The free CaO from the fly ash reacts with CO_2_ to form CaCO_3_. At temperatures ranging from 750 to 950 °C, the reversible reaction can lead to the decomposition of CaCO_3_ [29].

In order to calculate the content of CaCO_3_ and the CO_2_ uptake in the calcareous fly ash, thermogravimetric analysis (TGA) was conducted on the carbonated and non-carbonated calcareous fly ash. The CaCO_3_ content can be calculated using the following equation, Equation (1):

W_CaCO_3__ (wt.%) = W_CO_2__·(M_CaCO_3__/M_CO_2__) (1)

where W_CaCO_3__ is the mass percent of CaCO_3_ in the samples (fly ash before and after carbonation), W_CO_2__ is the mass loss from 570 to 940 °C during the TG measurements because of the decomposition of CaCO_3_, and M_CaCO_3__ and M_CO_2__ are the molar masses of CaCO_3_ and CO_2_, respectively [30].

The CO_2_ uptake can be estimated using the following equation, Equation (2) [31]:

CO_2_ uptake (%) = ((m_570°C_ − m_940°C_)/m_s_)·100 (2)

where m_570°C_ is the sample mass at 570 °C, m_940°C_ is the sample mass at 940 °C, and m_s_ is the mass of the sample.

In addition3, the CO_2_ uptake was measured through the mass gain of the fly ash sample after carbonation and was confirmed by the LOI results (Table 1).

#### 2.3.1. TGA

Thermogravimetric analysis was conducted on carbonated and non-carbonated calcareous fly ash. A SDT Q600 instrument (TA Instruments) DSC/TGA equipment, which provides a weighing accuracy of ±0.5% and a weighing precision of ±0.1%, was employed. About 20 mg of powder samples was mounted to an alumina crucible, which was heated from 20 °C up to 1000 °C at a ramping rate of 20 °C/min. The mass of the crucible and sample was continuously recorded during the heating process, in which the furnace was purged with pure N_2_ gas at a flow rate of 100 mL/min.

#### 2.3.2. XRD

The Bruker D8 Advance high resolution diffractometer was used to conduct the XRD test on the 25 mg powder samples. The source of the X-rays was generated through the Cu-Kα radiation, with diffraction patterns collected in the range of 10–80° 2θ at 0.02° per step.

#### 2.3.3. SEM

The surface of the samples was examined with a high-resolution scanning electron microscope (SEM) Prisma E (ThermoFisher, Waltham, MA, USA) using a secondary electron detector. Analyses were performed at accelerating voltage between 10 and 20 kV. The flat samples, measuring 12 mm in diameter and 6 mm in height, were coated with a 5 nm-thick layer of gold (Au). Observation was made at a 5000× magnification for fly ash samples and 2000× magnification for concrete specimens.

### 2.4. Mortar and Concrete Characterization

#### 2.4.1. Compressive Strength Test

The compressive strength of concrete specimens was studied after 2, 7, 28, 56, and 90 days of curing, while mortar specimens were tested after 28 and 56 days of curing. The compressive strength testing was carried out in accordance with PN-85/B-04500 [32] on the six halves of the beams remaining after the flexural strength test and in accordance with PN-EN 196-1 on three concrete cubic specimens with dimensions of 100 mm. Measurement was performed using a CONTROLS AUTOMAX (CONTROLS S.p.A, Milano, Italy) testing machine. The loading rate was 0.5 MPa/s.

#### 2.4.2. Determination of pH

The neutralizing impact was another significant measure of carbonation, as it had the ability to lower the pH within the samples. An aqueous extract was prepared from the crushed residue of mortar specimens after the compressive strength test. The mortar residue was mixed with water at a mass ratio of 1:1 and set for 24 h. The sample was then filtered, and the pH of the water extracts was determined by measuring with a pH meter (Methrom) and determining the pH from the titration. Prior to the test, the pH meter was adjusted using three distinct buffer solutions with pH levels of 7, 9, and 12.

#### 2.4.3. Resistance to Weathering Carbonation

Three concrete prisms and three mortar beams of each composition were cast and cured for 28 days in accordance with PN-EN 12390-2 [33]. The test specimens were conditioned in a laboratory air environment for 14 days and then placed into the carbonation chamber with a temperature of 21 ± 2 °C, RH 60 ± 10%, and 3% CO_2_ according to PN-EN 12390-12 [34]. One prism and beam of each composition was taken out of the carbonation chamber after 7, 28, 70, and 140 days of exposure. Right after the designated storage duration, the specimens were split, and the cross section was sprayed with a phenolphthalein solution. The depth of carbonation was measured at 12 locations on each concrete or mortar sample. In areas where carbonation did not affect the specimen (pH ranging from above 9.3), the indicator turned pink, while the carbonated surface remained uncolored. The outcomes were graphed against the square root of time, and the carbonation rate was determined by calculating the slope of the fitted line using the following equation, Equation (3):

d_k_ = a + K_AC_·√t (3)

where d_k_ (mm) is mean carbonation depth at time t (days), a (mm) is a constant representing the *y*-axis intercept, and K_AC_ (mm·day^−^^0.5^) is the carbonation rate [35].

#### 2.4.4. EIS Analysis

**Figure 2:**
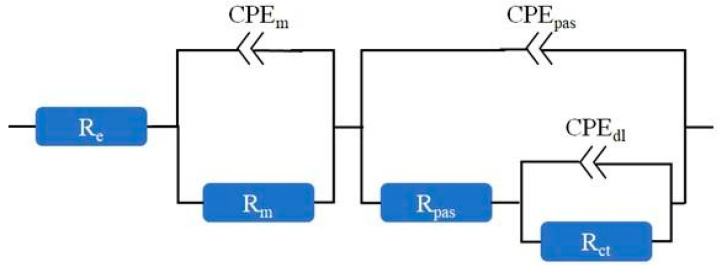
Equivalent circuit for steel in cement mortar.

After 28 days of curing in a climatic chamber (T = 20 ± 2 °C, RH = 90 ± 10%), reinforced mortars were placed in a measuring container filled with distilled water for 24 h. After this time, electrochemical impedance spectroscopy (EIS) measurement was carried out, followed by the measurement of the polarization curve of the steel reinforcement. These measurements were performed using the Autolab PGSTAT 302N potentiostat/galvanostat with Nova 2.1.6 software. The measurements were conducted in a three-electrode arrangement, in which the mortar reinforcement served as the working electrode. As a reference electrode, a saturated calomel electrode (SCE) was used. The counter electrode was a stainless-steel plate. EIS measurements were carried out at a corrosion potential in the frequency range of 50 kHz–3 mHz. The amplitude of the sinusoidal excitation signal was 10 mV. The obtained impedance spectra were analyzed using an electrical equivalent circuit presented in Figure 2. The circuit is an equivalent circuit as in the work [36] but without the Warburg (diffusional) impedance (W). The Warburg impedance was removed because the impedance spectra obtained did not indicate the presence of diffusion processes.

The following parameters describing the tested system were determined in this way: R_e_—the electrolyte resistance; R_m_—mortar resistance; CPE_m_—constant phase element describing mortar; R_pas_—the ohmic resistance in pits or defects of passive layer; CPE_pas_—the constant phase element for passive surface; R_ct_—the charge transfer resistance; and CPE_dl_—constant phase element describing the double layer on the steel. The polarization resistance, R_p_ (R_p_ = R_pas_ + R_ct_), which is inversely proportional to the corrosion rate, was also determined.

The polarization curves of the embedded rebar were recorded from a potential 0.15 V lower than the corrosion potential to a potential of 0.8 V in relation to SCE. The rate of change of the potential was 0.42 mV/s. From the polarization curve, the corrosion potential (E_cor_), the transpassive potential (E_tr_), and the passive current density at a potential of 0.244 V relative to SCE (j_p_) were read. Using the “corrosion rate” function in NOVA 2.1.6 software, the corrosion current density was determined. Additionally, the corrosion rate (CR) was calculated according to Equation (4):

CR = 0.0116j_cor_ (4)

where CR—corrosion rate (mm·year^−1^) and j_cor_—corrosion current density (μA·cm^−2^).

#### 2.4.5. The Diffusion of Chloride Ions in Concrete

The chloride ion diffusion tests were carried out on 150 × 150 × 150 mm concrete cubes. After 28 days of curing in water, the samples were cut in half. The cutting surface was perpendicular to the top surface of the samples relative to their original molded orientation. All the surfaces of the sample, except for the surface created by the cutting of the sample, were sealed with paraffin. The prepared samples were placed in a 3% NaCl solution. Chlorides penetrated into the sample through one unsealed paraffin surface (the ‘cut’ surface). After 55 days, the concrete cubes were removed from the NaCl solution, their surfaces were dried with a paper towel, and concrete samples were taken using a Profile Grinder kit. Samples for quantitative analysis of chloride ions were taken from the surface formed by cutting the cubes. The thickness of the collected layers of concrete samples was 1 mm. The collected material was dried at 105 °C, and its chloride ion content was determined using the Volhard method. The concrete samples were dissolved in water solution of nitric acid. The results obtained by this method represent the total chloride content, including both free and bound chlorides. For each composition of concrete, the test was performed on both halves of a split concrete cube. The chloride content in untreated concrete (c_i_) was also determined. The obtained distribution of chloride content in concrete was fitted to Equation (5) to determine the effective diffusion coefficient D_eff_ of chloride ions in concrete.

c = c_i_ + (c_0_ − c_i_) [1 − erf(x/(2·√(D_eff_·t)))] (5)

where c—concentration of chloride ions in concrete at depth x, c_0_—the concentration of chloride ions in the near-surface layer of concrete, c_i_—the initial concentration of chloride ions in concrete, erf—error function, x—distance from the surface of the concrete, and t—time of chloride penetration into the concrete.

#### 2.4.6. Service Life Reinforced Concrete Structures—Modeling

Chlorides penetrating into concrete cause a risk of corrosion of reinforcement, which limits the service life of reinforced concrete structures. Based on the obtained D_eff_ values (point 2.4.5), it is possible to estimate the service life of a structure made of such concrete exposed to chloride ions. It is important to also consider the effect of the structure’s ambient temperature on D_eff_ and the fact that D_eff_ decreases over time. The slower diffusion of chloride in older concrete is related to the sealing the concrete microstructure due to binder hydration and the binding of chloride ions into salts that crystallize with increasing volume [37,38]. The diffusion coefficient D_eff_ at any time can be estimated from Equation (6):

D_eff_ = D_eff0_·(t_0_/t)^m^ (6)

where D_eff0_ is the value of the effective diffusion coefficient determined from measurements at time t_0_ and m is the parameter characterizing the change in diffusion coefficient over time. The value of m depends primarily on the type of cement and w/c. For concretes made from Portland cement, the literature data indicate that the parameter m ranges from 0.1 to 0.34 [39]. For concretes containing fly ash, the values of the m parameter are higher due to the dilution effect, i.e., cement is diluted by the incorporation of fly ash, as well as the slower hydration process and the associated changes that cause the concrete structure to be sealed over time. Guidelines from the paper [40] recommend adopting a value of m = 0.3 for concrete with cement CEM I and m = 0.6 if the cement binder contained fly ash. In order to include the effect of temperature on D_eff_ under real conditions, Equation (7) is used:

D_eff_ = k_e_·D_eff0_·k_t_(t_0_/t)^m^ (7)

where k_e_ is a parameter describing the effect of ambient temperature on D_eff_ and k_t_ is a parameter for transferring test results to the actual situation in the structure [40]. The k_e_ parameter is calculated from Equation (8):

k_e_ = exp[b_e_((1/T_ref_) − (1/T_real_)] (8)

where T_ref_—the temperature at which the D_eff_ determination test is performed (T_ref_ = 293 K), T_real_—the average ambient temperature of the structure, and be is a variable whose average value is 4800 K [40].

## 3. Results and Discussion

### 3.1. CO_2_ Uptake and Carbonation Product

#### 3.1.1. TGA

**Figure 3:**
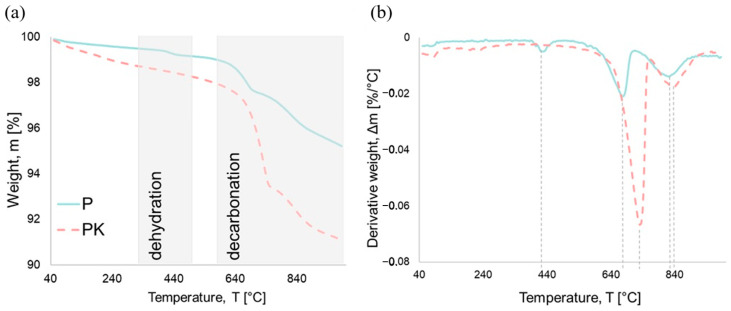
(**a**) TGA curves of calcareous fly ash (P—non-carbonated, PK—carbonated); (**b**) DTA curves of calcareous fly ash (peak temperatures are highlighted with dashed lines).

To access the chemical changes in the fly ash after CO_2_ sequestration process, a TG analysis was conducted on the fly ash before and after carbonation. Figure 3 illustrates the results of (a) thermogravimetric analyses (TGA) and (b) differential thermal analyses (DTA) obtained for both carbonated (PK) and non-carbonated calcareous fly ash (P). Within the range of room temperature to approximately 100 °C, the reduction in mass is primarily attributed to a decrease in moisture content [41]. After that, the TGA curves reveal two distinct temperature ranges (Figure 3a). The first range is approximately located between 320 and 500 °C, whereas the second is between 570 and 940 °C. The first range corresponds to a dehydration range associated with the loss of absorbed water in the range of 105 to 400 °C and the calcium hydroxide (CH) dehydration in the range of 400 to 500 °C [42,43,44]. The second range corresponds to the decarbonation process typically taking place between 530 and 900 °C [45,46,47]. The peaks in the DTA curves at the corresponding temperatures (Figure 3b) corroborate these results. The non-carbonated calcareous fly ash exhibits its dehydration peak at 430 °C and its decarbonation peak at 670 °C. In the case of carbonated fly ash, no peak caused by the dehydration of CH was observed. The decarbonation peak shifts to higher temperatures at 730 °C for the carbonated calcareous fly ash. The loss in mass at this temperature can also be explained by the decomposition of the CaCO_3_ present in fly ashes. The absence of a peak in the CH dehydration range and a sharper peak in the decarbonation range observed for the carbonated fly ash can be attributed to the reaction between CH and CO_2_ during sequestration process, as a result of which CaCO_3_ was formed. Using this information, the CaCO_3_ content and the CO_2_ uptake can be determined. From Equation (1), the total amount of CaCO_3_ in the PK ash was found to be 7.2%, while from Equation (2), the CO_2_ uptake was found to be 3.2%.

#### 3.1.2. XRD

**Figure 4:**
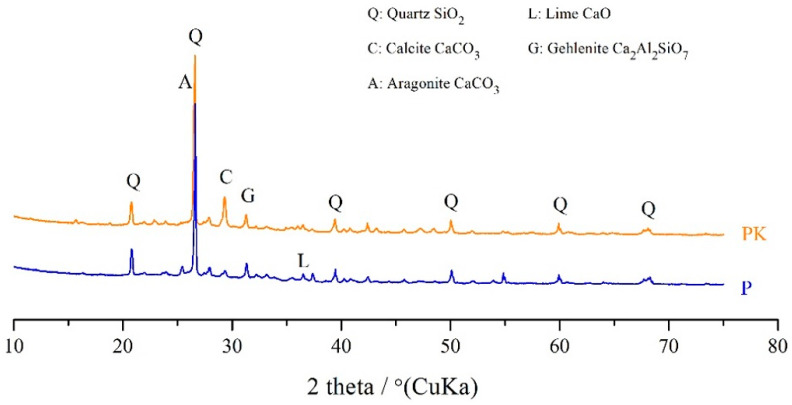
XRD spectra of the carbonated (PK) and non-carbonated (P) calcareous fly ash.

Figure 4 shows the XRD spectra of the calcareous fly ash before and after CO_2_ sequestration. The main crystalline mineral phases detected in the non-carbonated fly ash (P) were quartz (SiO_2_), lime (CaO), calcite (CaCO_3_), and brucite (Mg(OH)_2_). The presence of calcite in the conventional coal fly ash could be due to the surface reaction of carbon dioxide in air with calcium hydroxide during the storage of the ash [48]. The carbonated calcareous fly ash (PK) showed a higher concentration of calcite (appears at 2θ = 29°) compared to the raw ash (P), which is due to the carbonation process of fly ash, which effectively absorbs CO_2_ and generates stable CaCO_3_ [49,50,51,52].

#### 3.1.3. Mass Gain and LOI

From the CO_2_ uptake mass gain method, when comparing masses of the fly ash before and after carbonation, an average CO_2_ uptake of 4.2% was recorded. Likewise the 3.9% change in the loss on ignition (LOI) and the small changes in the compositions of carbonated calcareous fly ash (PK), compared to P fly ash, according to Table 1, was registered. The noticeable change in the LOI in the PK ash indicates a higher residual carbon content and a higher presence of carbonates [53,54], thus confirming the binding of CO_2_ in the ash.

#### 3.1.4. SEM

**Figure 5:**
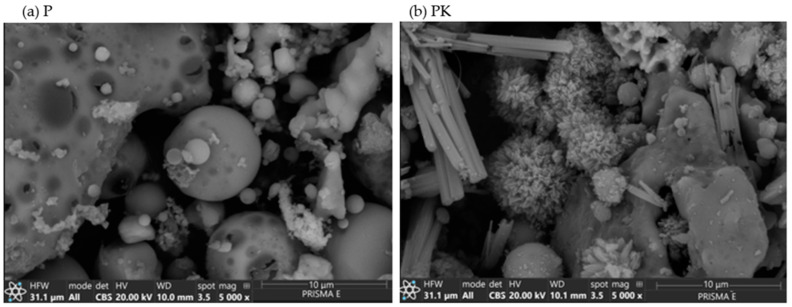
SEM image of (**a**) non-carbonated calcareous fly ash (P) and (**b**) carbonated calcareous fly ash (PK).

SEM analysis was used to determine the morphology of the calcareous fly ash before and after CO_2_ sequestration (Figure 5). The particle structure of calcareous fly ash (P) before CO_2_ sequestration was spherical but also contains flake particles (Figure 5a). After CO_2_ sequestration, the particle structure of the ash has changed. Needle-like crystals typical for aragonite could be observed [55,56], with a mixture of irregular polyhedrons and bundles [57].

**Figure 6:**
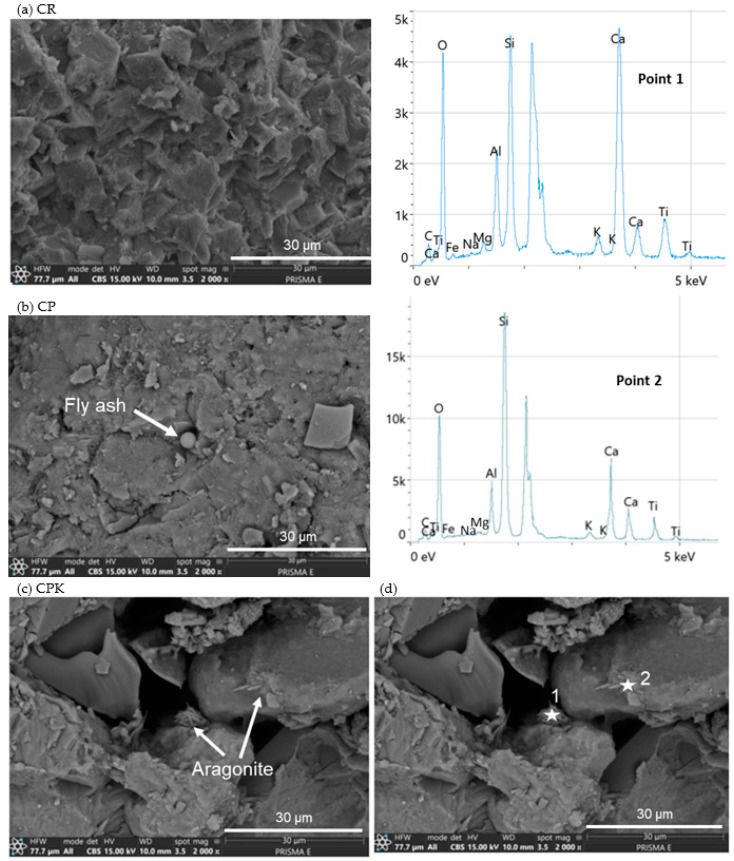
SEM of the concrete specimens (**a**) CR—reference concrete, (**b**) CP—concrete with non-carbonated calcareous fly ash, (**c**) CPK—concrete with carbonated calcareous fly ash, and (**d**) EDS from concrete with carbonated calcareous fly ash (CPK).

An SEM analysis was also used to determine the microstructure of the concrete when the non-carbonated (CP) and carbonated fly ash (CPK) was used (Figure 6). As can be seen from Figure 6a, there are plated-shape calcium hydroxide in the reference concrete (CR), C-S-H gel, and some pores also can be observed. Concrete with the non-carbonated calcareous fly ash (CP, Figure 6b) was characterized by a similar-to-the-aforementioned microstructure, but also, un-hydrated spherical particles of ash can be observed. However, the SEM observation of CPK concrete (Figure 6c) shows that the microstructure changes with the incorporation of carbonated calcareous fly ash (PK); the needle-like crystals typical for aragonite can be observed [55,56], supported by EDS analysis in Figure 6d.

The EDS patterns of the areas of interest of the CPK concrete are shown in Figure 6d. The surfaces are dominated by calcium, aluminum, silicon, oxygen, and carbon.

### 3.2. Mortar and Concrete Characterization

#### 3.2.1. Strength Development

**Figure 7:**
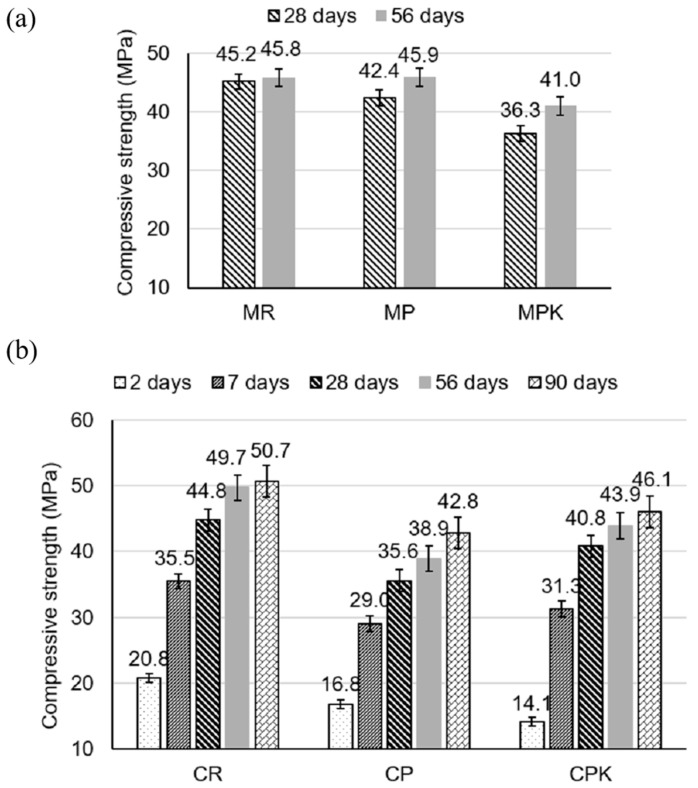
Compressive strength of (**a**) mortars and (**b**) concretes—MR/CR—reference batch, MP/CP—batch with non-carbonated calcareous fly ash, and MPK/CPK—batch with carbonated calcareous fly ash.

The evaluation of the viability of incorporating fly ash as a mineral additive requires the examination of compressive strength as an essential characteristic. Figure 7a illustrates the compressive strengths of mortars at different ages (28 and 56 days) where calcareous fly ash before and after CO_2_ sequestration were incorporated. Specimens containing calcareous fly ash, after 28 days of curing, were characterized by lower compressive strength compared to the reference mortar (cement-only mortar). Nevertheless, following a 56-day curing period, the compressive strength of the mortar with non-carbonated calcareous fly ash (MP) was restored to a level comparable to that of the cement-only mortar. Compared to the non-carbonated counterparts, the compressive strength of mortar specimens with carbonated calcareous fly ash (MPK) was lower by 14% after 28 days and 10% after 56 days. The mortar specimens containing carbonated fly ash necessitate a more prolonged duration compared to those solely composed of cement in order to achieve the desired level of compressive strength. The explanation lies in the fact that the pozzolanic reaction of fly ash takes a longer time for strength gain compared to cement hydration [58].

Figure 7b illustrates the compressive strengths of concretes at different ages (2, 7, 28, 56, and 90 days) where of calcareous fly ash before and after CO_2_ sequestration were incorporated. Specimens containing calcareous fly ash were characterized by lower compressive strength compared to the reference concrete (cement-only concrete). The compressive strengths of concrete specimens with carbonated calcareous fly ash (CPK) is on average 5.4% higher compared to their non-carbonated counterparts. Despite this difference in compressive strengths, it can be assumed that both types of specimens are in the same strength class.

Differences in the strength development of mortars and concretes containing carbonated and non-carbonated fly ash may result from several key factors related to their material properties. Carbonated fly ash has a lower content of oxides involved in the binding of the binder and a lower calcium content, which is essential for the formation of cement hydration products, such as calcium silicates (CSH) [59], resulting in an extended strength gain time for mortars employing it. Additionally, the binder content in the tested samples varies. In the case of mortars, the binder content is 25% *w*/*w*, while in concrete, it is significantly lower at 14.5% *w*/*w*. For mortars, carbonated fly ash may have a higher impact on the binding properties of the binder, negatively affecting the strength of the mortars. Conversely, in concretes, the effect of microstructural sealing by the carbonated fly ash may dominate over its negative impact on the binding properties of the binder, leading to a faster strength gain of concrete compared to mortars. Also in mortars, the lesser sealing effect of the fly ash on the microstructure may lead to higher porosity, which in turn affects the decrease in mortar strength [59].

A one-way ANOVA was conducted to compare the effect of cement substitution with calcareous fly ash on the compressive strength of mortar and concrete. The results showed that there was no statistically significant difference in compressive strength between the specimens both for mortar (*p* = 0.122) and concrete (*p* = 0.548).

While the reduction in concrete compressive strength observed following CO_2_ sequestration in calcareous fly ash is relatively minor (phi factor avg. 0.9), it is critical to assess its acceptability within the context of structural performance requirements. According to structural design codes and safety guidelines, a strength reduction depends on the specific application and is in the range of 0.6–0.9 [60]. To compensate the strength reduction, mix designs can be optimized (e.g., by adjusting the water-to-binder ratio or incorporating additional pozzolanic materials), chemical or mineral admixtures can be used to enhance matrix densification and early-age strength development, and controlled carbonation curing regimes can be implemented to maximize CO_2_ uptake without negatively affecting mechanical properties [61]. Moreover, emerging research suggests that fiber reinforcement may further improve tensile and flexural behavior in carbonated systems, offering another avenue for maintaining structural integrity [62].

#### 3.2.2. Evaluation of pH Values After CO_2_ Sequestration

The alkaline environment of mortars containing calcareous fly ash was examined on the aqueous mortar extracts both using a pH meter and through titration. The pH value of reference mortar was also measured for comparison. Table 3 shows the measured pH results. The replacement of 10% of cement with both non-carbonated and carbonated ash resulted in a decrease in the pH of the concrete pore solution of concrete. The concentration of OH- ions in MPK extracts was reduced by about 19% and 5% compared to MR and MP, respectively. The pH reduction can be associated with the conversion of free CaO into CaCO_3_ during fly ash carbonation. Smaller differences were observed for pH determination by titration, but it confirmed the relationship obtained from measuring pH using a pH meter. Thus, the introduction of 10 wt.% carbonated calcareous fly ash did not result in a severely decreased pH, thus eliminating the risk of destructing the passive layer on the embedded reinforcing steel. The obtained pH values above 12 are typical for cement-based concrete. In such an alkaline environment, steel reinforcement is protected by a passive oxide layer, and its corrosion rate is negligible (the reinforcement is considered non-corrosive). According to the ACI 222R-19 [63] standard, steel remains passive in concrete as long as the pH remains above approximately 11.5, with optimal passivation occurring at pH levels above 12.5. However, this is a simplification, as a reduction in pH alone does not always initiate corrosion of the reinforcement. The moisture content at the steel–concrete interface plays a critical role in influencing the corrosion rate of steel in concrete with reduced alkalinity [64].

#### 3.2.3. Weathering Carbonation

**Figure 8:**
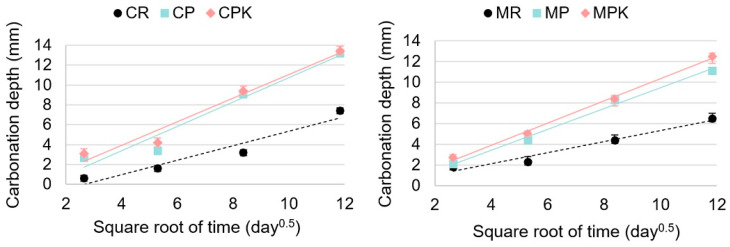
Carbonation depth of CR (MR)—reference concrete (mortar), CP (MP)—concrete (mortar) with non-carbonated calcareous fly ash, and CPK (MPK)—concrete (mortar) with carbonated calcareous fly ash.

The visual evaluation of carbonation depth was conducted by spraying the pH indicator onto the freshly split surfaces of the samples. The resulting data, depicting the relationship between carbonation depth and the square root of time, can be found in Figure 8. Generally, there exists a linear relationship between these two variables, as outlined in Table 4. The R^2^ values ranged from 0.86 to 0.99, indicating a good-to-excellent level of accuracy in the fitting process.

**Figure 9:**
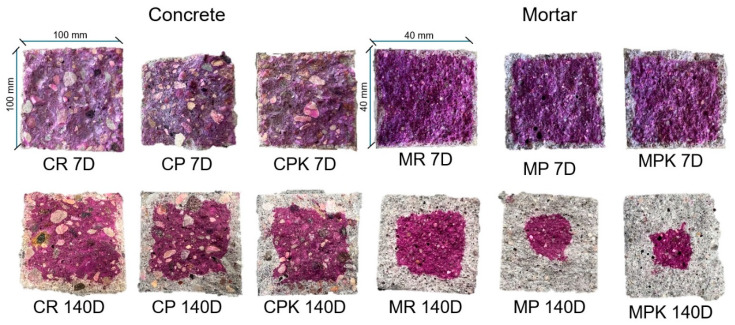
Carbonation fronts of specimens CR (MR)—reference concrete (mortar), CP (MP)—concrete (mortar) with non-carbonated calcareous fly ash, and CPK (MPK)—concrete (mortar) with carbonated calcareous fly ash after 7 and 140 days of carbonation.

The findings indicate that the carbonation rate observed in the concrete and mortar containing calcareous fly ash was higher compared to the reference specimens. The carbonation rate of concrete with carbonated and non-carbonated fly ash was similar (the difference was 4%). The effect of fly ash carbonation on the depth of concrete carbonation is statistically insignificant (*p* = 0.346). The carbonation tests conducted were accelerated, with a CO_2_ concentration of 3%, which is two orders of magnitude higher than the average atmospheric CO_2_ concentration (approximately 0.04%). Therefore, the results of these tests are indicative of the long-term impact of fly ash carbonation on concrete carbonation. After 140 days of exposure, the CPK demonstrated a carbonation depth of 13.4 mm and CP a carbonation depth of 13.2 mm, as opposed to the 7.4 mm measured on the CR. Similarly, the MPK demonstrated a carbonation depth of 12.5 mm and MP of 11.1 mm, while only 6.5 mm was measured on the MR (Figure 9). It is found that the carbonation rate of concrete increased with the addition of the fly ash [65,66] because of the lower amount of Ca(OH)_2_, delayed hydration, and increased porosity of the concrete [67,68]. However, when the concrete samples incorporate carbonated calcareous fly ash in the binder, the carbonation rate is slightly lower compared to their counterparts with the non-carbonated ash. This might be related to the change in the pore structure. The CaCO_3_ in the carbonated fly ash may promote the stability of ettringite in the blended cement. Furthermore, it is presumed that CaCO_3_ could react with the aluminate phase in C_3_A and C_4_AF to form carboaluminate [69,70,71]. The formation of the above-mentioned compounds can densify the microstructure, which results in the slowing down of the movement of the carbonation front [72,73].

#### 3.2.4. Electrochemical Measurements Analysis

**Figure 10:**
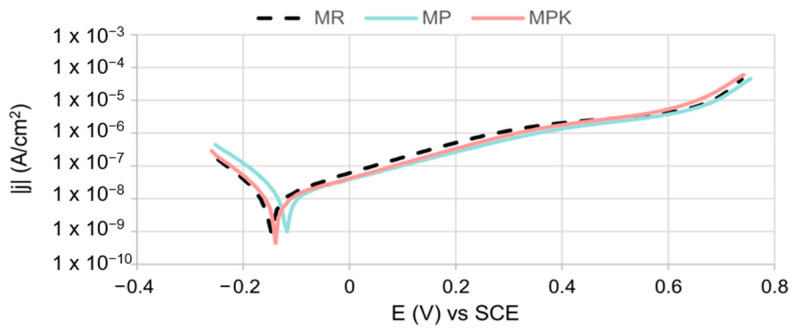
Polarization curves of steel in mortars MR—reference mortar, MP—mortar with non-carbonated calcareous fly ash, and MPK—mortar with carbonated calcareous fly ash.

Figure 10 presents the anodic polarization curves of the embedded steel in the tested mortars. The course of reinforcement polarization curves in all three types of mortars was very similar to each other, indicating a lack of significant influence of the additives used on the corrosion process of reinforcement in mortars. The corrosion potential was −0.149 V for steel in MPK and MR mortars. The corrosion potential of the reinforcement in the MP mortar is slightly shifted towards higher potentials. The corrosion potential values of all specimens meet the requirements for steel in a passive state according to the PN-B-01810 [74] standard. The anodic current density was very low in all cases over a wide range of potentials (a rapid increase in anodic current density was observed only at a potential of approximately 0.66 V). Such a course of polarization curves confirms the passivation of steel in mortars.

**Figure 11:**
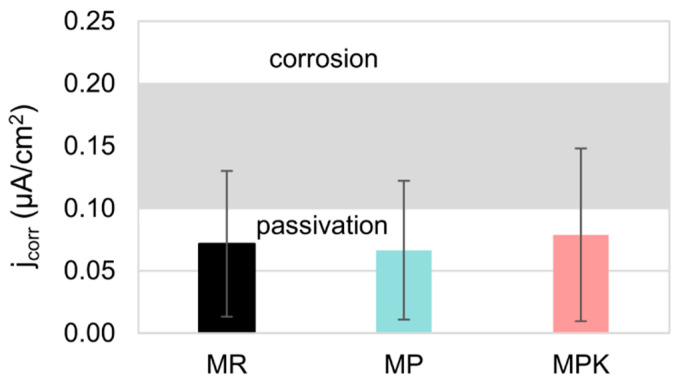
The influence of cement substitution with calcareous fly ash on the corrosion current density of mortar reinforcement MR—reference mortar, MP—mortar with non-carbonated calcareous fly ash, and MPK—mortar with carbonated calcareous fly ash.

The parameters (*E*_cor_, *j*_cor_, *j*_p_, *E*_tr_) determined based on the analysis of polarization curves meet the requirements for the passive state of steel according to the PN-B-01810 standard and Andrade [75] (Table 5). Steel has remained passivation in all tested mortars. The *j*_cor_ steel values in all mortars are low and similar to each other, indicating negligibly small corrosion and depassivation rates of the steel (Figure 11) as well as the absence of significant impact of the additives used on the rate of reinforcement corrosion. The corrosion rate values calculated based on the *j*_cor_ values are very low (<0.001 mpy), which indicates the overall corrosion resistance of the reinforcement in the tested mortars according to the PN-H-04608 [76] standard. All polarization curves were characterized by low *j*_p_ values, indicating very good protective properties of the passive layer. It can be observed that the value of *j*_p_ for steel in the MP mortar is lower compared to other mortars, indicating a slight improvement in the protective properties of the passive layer by the non-carbonated calcareous fly ash. Also in work [77], no negative effect of cement containing calcareous fly ash on the steel reinforcement in concrete was found.

**Figure 12:**
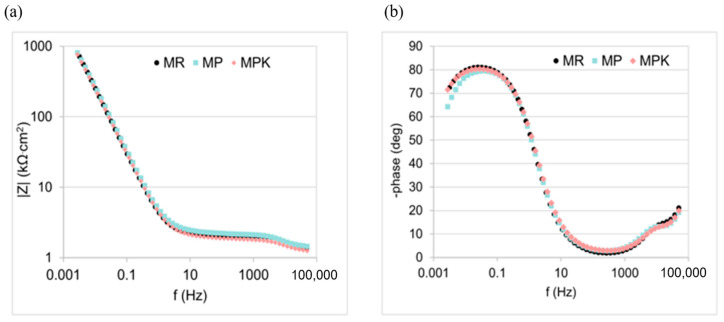
EIS spectra: (**a**) impedance, (**b**) phase shift for steel in mortars MR—reference mortar, MP—mortar with non-carbonated calcareous fly ash, and MPK—mortar with carbonated calcareous fly ash.

The course of impedance spectra for the reinforcement in the tested mortars was very similar to each other (Figure 12). All spectra were characterized by high impedance values at low frequencies, indicating a high polarization resistance of the reinforcement and thus a low corrosion rate. All spectra were capacitive in nature (a broad peak of the phase shift angle at low frequencies), characteristic of steel in the passive state [78]. The values of the phase shift angle at low frequencies were high and indicated good protective properties of the passive layer on the steel.

Equivalent circuit parameters for steel in the tested mortars determined from the analysis of impedance spectra are presented in Table 6. A good fit of the obtained spectra to the applied electrical equivalent circuit was achieved (χ^2^ in the range of 0.01–0.02). The estimated error—calculated by testing marginal variations of the fitted or calculated values near convergence—for the *R*_ct_ parameter (the most important parameter for determining the corrosion rate) ranged from 5% to 15%. The determined values of R_ct_ in all cases are very high and close to each other, which indicates a very low corrosion rate. In corrosion studies of heterogeneous systems like steel in concrete, large standard deviations of charge transfer resistance values (or corrosion current, which is inversely proportional to charge transfer resistance) are often observed and can even be considered typical [78,79]. This is related to the heterogeneity of the steel surface as well as the heterogeneity of the steel–concrete transition zone. All charge transfer resistance values were >500 kΩ·cm^2^, indicating a passive state of the steel [78].

The Y_pas_ parameter is small, and n_pas_ values are close to 1, indicating a thin, homogeneous passive layer on the steel with very good protective properties [78]. The Y_dl_ parameter is small, and the n_dl_ parameter is high, indicating a small active area and a homogeneous double layer [78]. Conclusions from the analysis of impedance spectra are compatible with those from the analysis of polarization curves: the steel in the tested mortars was in a passive state, and no significant effect of the applied additives on the corrosion of the reinforcement was observed.

#### 3.2.5. The Diffusion of Chloride Ions in Concrete

**Figure 13:**
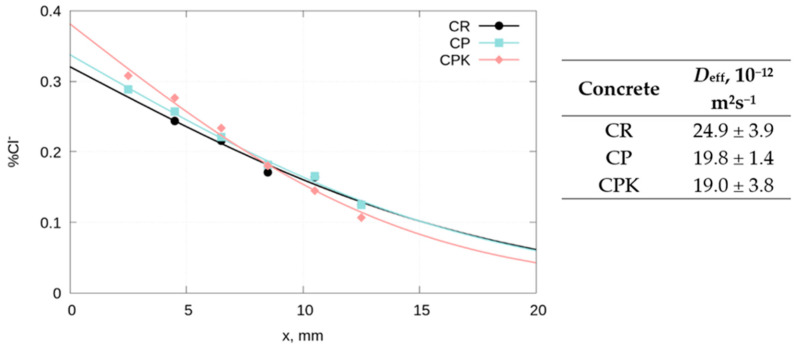
The distribution of chloride ion content in concrete fitted to Equation (5) depending on the concrete sample used (CR—reference concrete, CP—concrete with non-carbonated calcareous fly ash, and CPK—concrete with carbonated calcareous fly ash).

The resulting distribution of chloride ion content in concrete fitted to Equation (5) is shown in Figure 13. The chloride content of untreated concrete was 0.013%. The effective diffusion coefficient of chlorides in concrete with calcareous fly ash (CP and CPK) is lower than the value obtained for the reference concrete CR (Figure 13), which may be related to the chemical composition of the hardened cement paste and its ability to bind chlorides. The ash used in this study has a higher Al_2_O_3_ content than cement (Table 1), resulting in the formation of more hydrated aluminates in concrete with ash. Chlorides penetrating into concrete react with hydrated calcium aluminates to form Friedel (3CaO·Al_2_O_3_·CaCl_2_·10H_2_O) and Kuzel (3CaO·Al_2_O_3_·0.5CaCl_2_·0.5CaSO_4_·10H_2_O) salts [37,38]. The increased content of hydrated aluminates in concrete with ash causes an increase in chloride binding capacity, which in turn reduces the rate of chloride ingress and seals the concrete. The effect of these phenomena is to reduce the effective diffusion coefficient of chlorides in concrete with ash. No significant influence of carbonated calcareous fly ash on the chloride diffusion rate was observed.

### 3.3. Service Life of Reinforced Concrete Structures—Modeling

Assuming conditions of the Polish Baltic coast and using Formulas (5), (7), and (8), the study estimated the time to reach the permissible chloride concentration at a depth of 40 mm (t_crit_) as well as the required cover thickness to provide 50-year and 100-year protection for the reinforcement (x_0.4% for 50 years_, x_0.4% for 100 years_). The permissible chloride concentration was taken as 0.4% by cement mass. This is the permissible chloride content for reinforced concrete according to PN-EN 206 [80]. For the tested concretes containing 300 kg of cement in 1 m^3^ and a density of 2300 kg/m^3^, a chloride content of 0.4% by cement mass corresponds to 0.052% of the concrete (%Cl^−^_crit_). The depth of 40 mm was adopted because of the requirements for the minimum thickness of concrete cover in exposure classes XD and XS, which is just 40 mm according to the standard PN-EN 206. Fifty years is the design life of general construction objects; in contrast, one hundred years is the design life of monumental building structures, bridges, and other engineering structures, among other hydrotechnical structures according to PN-EN 1990:2004/A1:2008 [81]. Calculations were performed for m equal to 0.3 for CR concrete, and for CP and CPK concretes, m equal to 0.6 is recommended by [40]. Assuming an average annual water temperature in the Baltic Sea of 10 °C, k_e_ = 0.56 was determined. We assumed c_0_ = 0.9% by cement mass (equivalent to 0.117% of the mass of concrete tested) [40] and k_t_ = 1, as in the paper [82]. The purpose of the present calculations was to illustrate the effect of D_eff_ values on the estimation of structural life and minimum concrete cover thickness under seawater immersion conditions. The prediction of the structural service live and minimum concrete cover thickness depends on the assumed parameter values and boundary conditions. In this study, the seawater temperature was assumed to be constant—no consideration was given to changes in temperature over time due to climate warming. An increase in temperature over time accelerates the rate of chloride diffusion. Time-dependent changes in the effective diffusion coefficient, D_eff_, are characterized by the parameter m, which was adopted in accordance with the recommendations in [40]. However, in practice, different concretes may exhibit slightly different values of m, and long-term experimental studies would be necessary to determine this parameter for a specific concrete composition. Changing the value of m significantly affects the predicted durability of the concrete—higher values of m correspond to slower chloride diffusion. The assumption of Cl^−^_crit_ = 0.4% by mass of cement, according to PN-EN 206, is also a simplification since the critical chloride content in concrete at which reinforcement corrosion is initiated depends on several factors, including the composition of the binder and the chloride-binding capacity of the hardened cement matrix (chlorides chemically bound by the cement matrix do not pose a corrosion risk to the reinforcement).

Given all these variables, predicting the durability of reinforced concrete structures is complex and inherently uncertain. The calculated values should therefore be regarded as estimates.

**Figure 14:**
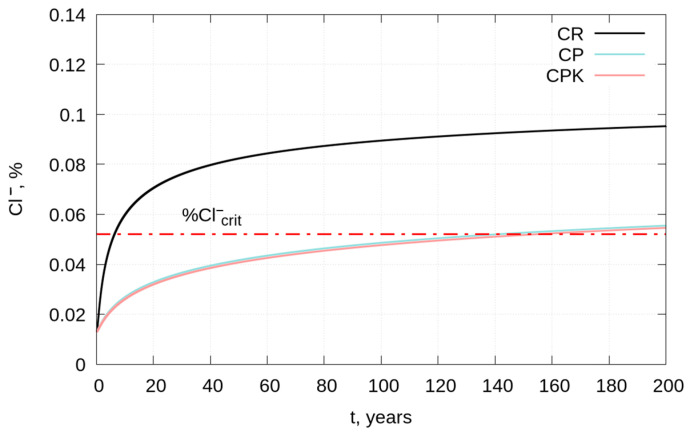
The dependence of chloride content in concrete (at 40 mm depth) on the time of chloride diffusion (CR—reference concrete, CP—concrete with non-carbonated calcareous fly ash, and CPK—concrete with carbonated calcareous fly ash). Red dotted line represents %Cl^−^_crit_ = 0.052.

The predicted chloride contents of concrete at a depth of 40 mm are significantly lower for concretes with ash (CP and CPK) than for the reference concrete (Figure 14). Changes in the chloride content of CP and CPK concretes at a depth of 40 mm over time are very similar to each other—the curves almost coincide. The estimated time to reach the permissible chloride concentration at a depth of 40 mm for CR concrete is very short and is about 6.3 years. The t_crit_ value for CP and CPK concretes is more than 20 times longer and is about 141 and 154 years, respectively. The significantly longer predicted life of CP and CPK concrete structures is related to the lower D_eff_ value for CP and CPK concretes and also to the predicted higher effect of time on D_eff_ changes (parameter m = 0.6).

The minimum cover thickness for the 50-year protection of reinforcement is 77.6 mm for CR concrete. Replacing 10% of cement with fly ash results in a nearly 3-fold reduction in the minimum cover thickness. No significant effect of ash carbonation was observed on the modeling results. For structures with an assumed lifespan of 100 years, the minimum cover thickness of CR concrete should be as much as 108 mm, while for CP and CPK concretes, it should be 39.8 and 39.1 mm, respectively (Table 7). The determined values of the cover to ensure protection of reinforcement against corrosion in CR concrete exceeded the value of the minimum thickness of the cover according to the requirements of PN-EN 206 for exposure classes XD and XS.

### 3.4. Scaling and Environmental Assessment

The scalability of CO_2_ sequestration processes is critical when considering their integration into industrial applications. While the carbonation process used in this study (5 bar CO_2_ pressure for 24 h) has demonstrated effectiveness in enhancing CO_2_ uptake and influencing material properties, its scalability to industrial levels warrants further discussion. Operating at elevated pressures and maintaining controlled environments over extended durations may pose economic and logistical challenges in large-scale applications, particularly in conventional concrete production or waste treatment facilities. However, prior studies have shown that pressurized carbonation can be scaled under certain conditions, especially when integrated into precast production lines, where curing chambers and controlled CO_2_ environments already exist [5,83]. Alternatively, optimizing parameters such as shorter curing durations, moderate pressure regimes, or recycling CO_2_ streams from industrial emissions could enhance both feasibility and sustainability [84]. A techno-economic assessment or life-cycle analysis could further support the viability of implementing this process at scale, ensuring that the carbon sequestration benefits are not offset by high energy or infrastructure costs.

While the present study emphasizes the technical viability of CO_2_ sequestration using calcareous fly ash, contextualizing the sequestration capacity in terms of national emission targets or construction-sector demand would underscore its environmental relevance. For example, if scaled across a fraction of national concrete production, even a modest sequestration rate (e.g., 0.1–0.2 kg CO_2_/kg ash) could result in the permanent storage of thousands to millions of tons of CO_2_ annually depending on fly ash availability and adoption rates [85]. Given that concrete production contributes approximately 7% of global CO_2_ emissions, integrating carbonation-curing or CO_2_-sequestering materials could play a non-trivial role in national decarbonization strategies—particularly in countries with significant coal combustion residues or cement usage [86]. A quantitative estimate of potential CO_2_ offset—linked to regional fly ash supply chains or projected infrastructure growth—would provide valuable insight into the scalability and climate mitigation potential of the proposed approach. Applying a sequestration rate of approximately 0.2 kg CO_2_ per kg of calcareous fly ash, large-scale implementation in Poland—where annual fly ash generation is around 4–5 million tons—could enable the permanent storage of up to 300,000 tons of CO_2_ per year, assuming 50% utilization. This is roughly equivalent to the annual emissions of 65,000 passenger vehicles, highlighting the potential contribution of this method to national climate goals. At a broader European scale, where fly ash availability remains substantial despite ongoing coal phaseouts, the utilization of 10 million tons of calcareous ash for CO_2_ sequestration could result in offsets of up to 2 million tons of CO_2_ annually. These figures underscore the feasibility of integrating mineral carbonation into regional carbon reduction strategies, particularly when aligned with construction material reuse and circular economy frameworks.

## 4. Conclusions

In summary, around 3% of CO_2_ was successfully stored in the calcareous fly ash using the presented method of CO_2_ sequestration. The innovative aspect of this study lies in its utilization of the carbonated calcareous fly ash in concrete and in particular reinforced mortar. A thorough analysis was conducted to examine the properties and behavior of mortar and concrete that incorporated the carbonated fly ash (10 wt.% of cement mass), and the following conclusions were obtained.

(1) The compressive strength of specimens with carbonated calcareous fly ash was lower compared to the reference samples; however, it was not more than 10% after 56 days for mortars and 9% after 90 days for concrete specimens.
(2) The introduction carbonated calcareous fly ash did not result in a severely decreased pH. The pH value of the water extract from the mortar did not drop below 12.
(3) It was found that the carbonation rate of concrete increases with the addition of the fly ash. However, no statistically significant effect (*p* > 0.05) of ash carbonation on concrete and mortar carbonation was observed.
(4) There was no statistically significant effect of fly ash, either carbonated or non-carbonated, on the corrosion behavior of steel in mortar. The course of reinforcement polarization curves in all tested types of mortars was very similar to each other, indicating steel passivation and good protective properties of the passive layer formed on the steel.
(5) Replacing 10% of the cement by mass with fly ash, both carbonated and non-carbonated, reduces the effective diffusion coefficient of chlorides in the concrete. No statistically significant influence of the carbonation of calcareous fly ash on the chloride diffusion rate was observed.
(6) The estimated time to reach the permissible chloride concentration at a depth of 40 mm for concretes with fly ash and with carbonated fly ash is more than 20 times longer compared to the reference concrete and is about 141 and 154 years, respectively.

While the current study has provided valuable insights into the potential for CO_2_ sequestration using calcareous fly ash, several important avenues for future research should be explored to enhance the applicability of this method in real-world construction contexts. One key area for future work is the investigation of higher substitution levels of fly ash in cementitious systems. While moderate substitution has been shown to provide effective CO_2_ sequestration without significant strength reductions, higher levels of fly ash incorporation may alter hydration kinetics, potentially reducing the early-age strength or modifying the long-term mechanical properties of the material. Research should focus on identifying the optimal substitution levels that balance both CO_2_ sequestration potential and structural performance while also considering long-term durability. Concrete materials made with calcareous fly ash should be tested under real-world exposure conditions to evaluate their durability and resilience in aggressive environments. Areas subject to freeze–thaw cycles can cause cracking and deterioration due to the expansion of water within the pore network. Accelerated testing methods, such as salt-water immersion, freeze–thaw cycling, and wet–dry cycling, will provide valuable insights into how carbonated fly ash-based concretes perform under these stressors. Long-term studies that track both carbonation depth and microstructural changes (e.g., pore structure, cracking, and phase transitions) will help to understand the potential trade-offs between continued sequestration and material durability. Pilot-scale testing will also allow for the evaluation of the economic feasibility of large-scale deployment and provide data on supply chain logistics for sourcing and distributing fly ash in carbon sequestration applications. By addressing these future research directions, the potential for calcareous fly ash as a CO_2_ sequestration method can be further optimized, ensuring its integration into both sustainable building practices and large-scale industrial applications.

## Figures and Tables

**Table 1:** Chemical composition (%) of tested materials.

Composition	SiO_2_	Al_2_O_3_	Fe_2_O_3_	CaO	MgO	SO_3_	K_2_O	P_2_O_5_	Rest	LOI
P	50.7	18.5	3.8	17.6	1.0	n.a	0.1	0.1	1.9	6.4
PK	48.5	17.7	3.7	17.0	0.9	n.a	0.1	0.1	1.7	10.3
CEM	17.5	5.3	3.7	63.8	1.7	4.8	1.3	0.3	0.4	1.2

Note: non-carbonated calcareous fly ash (P), carbonated calcareous fly ash (PK), and cement CEM I 42.5R (CEM).

**Table 2:** Compositions of prepared specimens.

Matrix	Cement, kg/m^3^	Fly Ash, kg/m^3^	Water, kg/m^3^	Gravel, kg/m^3^	Sand, kg/m^3^	w/c	sp/c, %
MR	512	-	256	-	1535	0.5	-
MP	461	51		-			-
MPK	461	51		-			-
CR	330	-	165	1931	-		1
CP	297	33			-		
CPK	297	33			-		

Note: MR/CR—reference mortar/concrete, MP/CP—mortar/concrete with non-carbonated calcareous fly ash, MPK/CPK—mortar/concrete with carbonated calcareous fly ash, sp—superplasticizer.

**Table 3:** pH values of mortar extracts (m_mortar_/m_water_ = 1:1, T = 25 °C).

Mortar	pH (pH Meter)	pH (Titration)	
		C_OH-_, M	pH
MR	12.57	0.0540	12.73
MP	12.46	0.0460	12.66
MPK	12.30	0.0435	12.64

Note: MR—reference mortar, MP—mortar with non-carbonated calcareous fly ash, MPK—mortar with carbonated calcareous fly ash.

**Table 4:** The results of the regression analysis conducted using the observed measurements of carbonation depth at four distinct time periods for CR (MR)—reference concrete (mortar), CP (MP)—concrete (mortar) with non-carbonated calcareous fly ash, and CPK (MPK)—concrete (mortar) with carbonated calcareous fly ash.

Parameter	CR	CP	CPK	MR	MP	MPK
K_AC_ (mm·day^−0.5^)	0.73	1.23	1.19	0.54	1.01	1.07
R^2^	0.93	0.95	0.97	0.97	0.99	0.99

**Table 5:** Characteristic parameters of polarization curves (1) according to PN-B-01810:1986 [74] and (2) according to Andrade [75]).

Parameter	MR	MP	MPK	Requirements for Passive State
*E*_cor_, V	−0.149 ± 0.012	−0.135 ± 0.023	−0.149 ± 0.013	>−0.350 ^(1)^
*j*_cor_, μAcm^−2^	0.072 ± 0.058	0.066 ± 0.056	0.079 ± 0.069	<0.1 ^(2)^
*j*_p_, μAcm^−2^	0.714 ± 0.119	0.440 ± 0.054	0.704 ± 0.257	<15 ^(1)^
*E*_tr_, V	0.660 ± 0.010	0.660 ± 0.010	0.660 ± 0.010	0.450–0.700 ^(1)^
*CR,* mpy	0.0008	0.0008	0.0009	<0.0016 ^(2)^

**Table 6:** Equivalent circuit parameters for steel in mortar determined from the analysis of impedance spectra.

Mortar	CPE_pas_		*R*_pas_, kΩ·cm^2^	CPE_dl_		*R*_ct_, kΩ·cm^2^
	*Y*_pas_, μFs^n−1^cm^−2^	*n*_pas_		*Y*_dl_, μFs^n−1^cm^−2^	*n*_dl_	
MR	34 ± 7	0.95 ± 0.01	2.5 ± 2.2	20 ± 6	0.89 ± 0.03	3620 ± 1410
MP	27 ± 3	0.95 ± 0.03	1.8 ± 1.3	22 ± 8	0.86 ± 0.01	3620 ± 1270
MPK	34 ± 1	0.94 ± 0.01	3.4 ± 1.5	15 ± 3	0.87 ± 0.01	3310 ± 1380

**Table 7:** Minimum thickness of concrete cover to ensure protection of reinforcement from chloride-initiated corrosion.

Concrete	CR	CP	CPK
x_0.4%, 50 years_, mm	76.6	28.1	27.6
x_0.4%, 100 years_, mm	108.0	39.8	39.1

## Data Availability

The original contributions presented in this study are included in the article. Further inquiries can be directed to the corresponding author.
